# The Human Factor in Industry

**Published:** 1921-03-12

**Authors:** 


THE PUBLIC WELL-BEING—(*continued*).

### The Human Factor in Industry.

The Duke of York, in his admirable speech at the Mansion House to the Industrial Welfare Society, did well to lay emphasis on the importance of the human factor in industry and to give encouragement to the work of the society in its efforts to aid the "renaissance of the spirit of industrial comradeship." This organisation has already done a real service to humanity in grappling with one of the most serious and far-reaching problems of modern industry—the threat which industrial units of immense size level against the individual worker, both as to his physical development and his spiritual outlook. It is a great point gained that science is now working hand in hand with the practical benevolent methods of voluntary social reformers to diminish industrial fatigue, or to reduce it to a minimum, and to improve the lot of the manual labourer in a score of ways by the application of a carefully devised system of humane administration.

Already many important facts relevant to this problem have been established. Thus it is an accepted maxim that a maximum of health is necessary for a maximum of productivity. Professor E. L. Collis, of the Welsh National School of Medicine, has shown how newly engaged workers suffer an undue proportion of lost time and sickness. Labour turnover—that is to say, the drift of labour from one place to another—is enormous, representing a serious economic loss which, on a conservative estimate, may be placed at £80,000,000 a year. This loss can be, and in certain cases has been, reduced by 65 per cent. Then it has been found that certain conditions of temperature represent the optimum for physical work, and that at the optimum temperature the greatest output and the fewest accidents occur. But monotony in temperature must be avoided; and, similarly, variations in relation to ventilation are important, for air should be constantly moving to give a maximum amount of stimulation. Monotony must also be eliminated in relation to the continuance of work, and for this purpose rest-pauses are invaluable, as by their proper use output may be increased in suitable cases, and over-fatigue, with its accompanying ill-being, prevented. Professor Collis further lays stress on the food factor, which has recently been closely investigated. It is now known that workmen doing heavy work require more food than those doing light work or brain work. Not only do they require more food, but they require more vitamines; and advantage is gained by providing means for the energy supply to be consumed under attractive surroundings.

We have mentioned these few broad discoveries, which may seem to relate only to the material side of human efficiency, mainly in order to show that scientific industrial research is moving towards the same goal, though by a different direction, as that aimed at by the social reformers; because, if such adjustments as are here indicated can be brought about, they will manifestly benefit not only the worker but the employer, by economising energy and increasing output. It becomes, therefore, the interest of both Capital and Labour frankly to co-operate in the dual task of perfecting labour-saving methods on the one hand, and on the other of improving the welfare of the operative and increasing his "joy of life." We believe there is a happy future for British industry if the vital question of the "human factor" is approached from this standpoint, and in this spirit. There is admittedly an urgent need at the present moment for greater efficiency and productivity, but what is needed still more urgently—for it is the predicate of permanent stability—is contentment among the mass of workers; and that is only to be realised by a firm and imaginative understanding between masters and men. That is what is meant when we express the longings of the nation for the creation of "a new spirit in industry."

